# South Africa and the Surgical Diaspora—A Hub for Surgical Migration and Training

**DOI:** 10.1007/s00268-023-06990-x

**Published:** 2023-04-08

**Authors:** Niclas Rudolfson, Adam Lantz, Mark G. Shrime, Walter Johnson, Martin D. Smith, Lars Hagander

**Affiliations:** 1 Surgery and Public Health, Pediatrics, Department of Clinical Sciences Lund, Lund University, Lund, Sweden; 2 Department of Urology, Kristianstad Central Hospital, Kristianstad, Sweden; 3 Department of Orthopedic Surgery, Helsingborg Hospital, Helsingborg, Sweden; 4 Mercy Ships, Garden Valley, TX USA; 5 Department of Global Health and Social Medicine, Harvard Medical School, Boston, MA USA; 6 Center for Global Surgery, Loma Linda University, Loma Linda, CA USA; 7 Department of Surgery, University of Witwatersrand, Johannesburg, South Africa; 8 Department of Pediatric Surgery, Skane University Hospital, Lund, Sweden

## Abstract

**Background:**

The shortage of trained surgeons, anesthesiologists, and obstetricians is a major contributor to the unmet need for surgical care in low- and middle-income countries, and the shortage is aggravated by migration to higher-income countries.

**Methods:**

We performed a cross-sectional observational study, combining individual-level data of 43,621 physicians from the Health Professions Council of South Africa with data from the registers of 14 high-income countries, and international statistics on surgical workforce, in order to quantify migration to and from South Africa in both absolute and relative terms.

**Results:**

Of 6670 surgeons, anesthesiologists, and obstetricians in South Africa, a total of 713 (11%) were foreign medical graduates, and 396 (6%) were from a low- or middle-income country. South Africa was an important destination primarily for physicians originating from low-income countries; 2% of all surgeons, anesthesiologists, and obstetricians from low- and middle-income countries were registered in South Africa, and 6% in the other 14 recipient countries. A total of 1295 (16%) South African surgeons, anesthesiologists, and obstetricians worked in any of the 14 studied high-income countries.

**Conclusion:**

South Africa is an important regional hub for surgical migration and training. A notable proportion of surgical specialists in South Africa were medical graduates from other low- or middle-income countries, whereas migration out of South Africa to high-income countries was even larger.

## Introduction

One of the most prominent barriers to surgical care is the acute shortage and maldistribution of trained surgeons, anesthesiologists, and obstetricians (SAO specialists) in low- and middle-income countries [1, 2], exacerbated by migration of these individuals to more affluent countries [3]. International health workforce mobility is often beneficial for training purposes, and professionally rewarding individually, yet higher-income countries’ dependency on SAO workforce from lower-income settings maintains the current maldistribution and prevents countries from strengthening self-sustainable domestic health systems of surgical care [4–9].

South Africa has previously been described as an exporter of well-trained physicians to high-income countries [4, 6, 10, 11], but while physicians from South Africa indeed migrate to high-income countries, South Africa also acts as a recipient of trainees and specialists from sub-Saharan countries [10, 12–16]. Consequently, South Africa has been described as an African hub of physician migration [4, 17–19]. However, the migration patterns of SAO specialists to and from South Africa have previously not been well characterized. Therefore, the objectives of this study were to quantify the current number of foreign medical graduates in surgery, anesthesia, and obstetrics, both immigrating from low- and middle-income countries to South Africa and emigrating from South Africa to high-income countries, and to estimate the proportion relative to the total SAO workforce from the perspective of both sending and receiving countries.

## Methods

### Study design

We performed a cross-sectional observational study of surgeons, anesthesiologists, and obstetricians licensed to practice in South Africa and compared this cadre with the WHO global surgery workforce database [20] and with more detailed workforce data of SAO specialists working in 14 major high-income countries.

### SAO specialists in South Africa

**Fig. 1:**
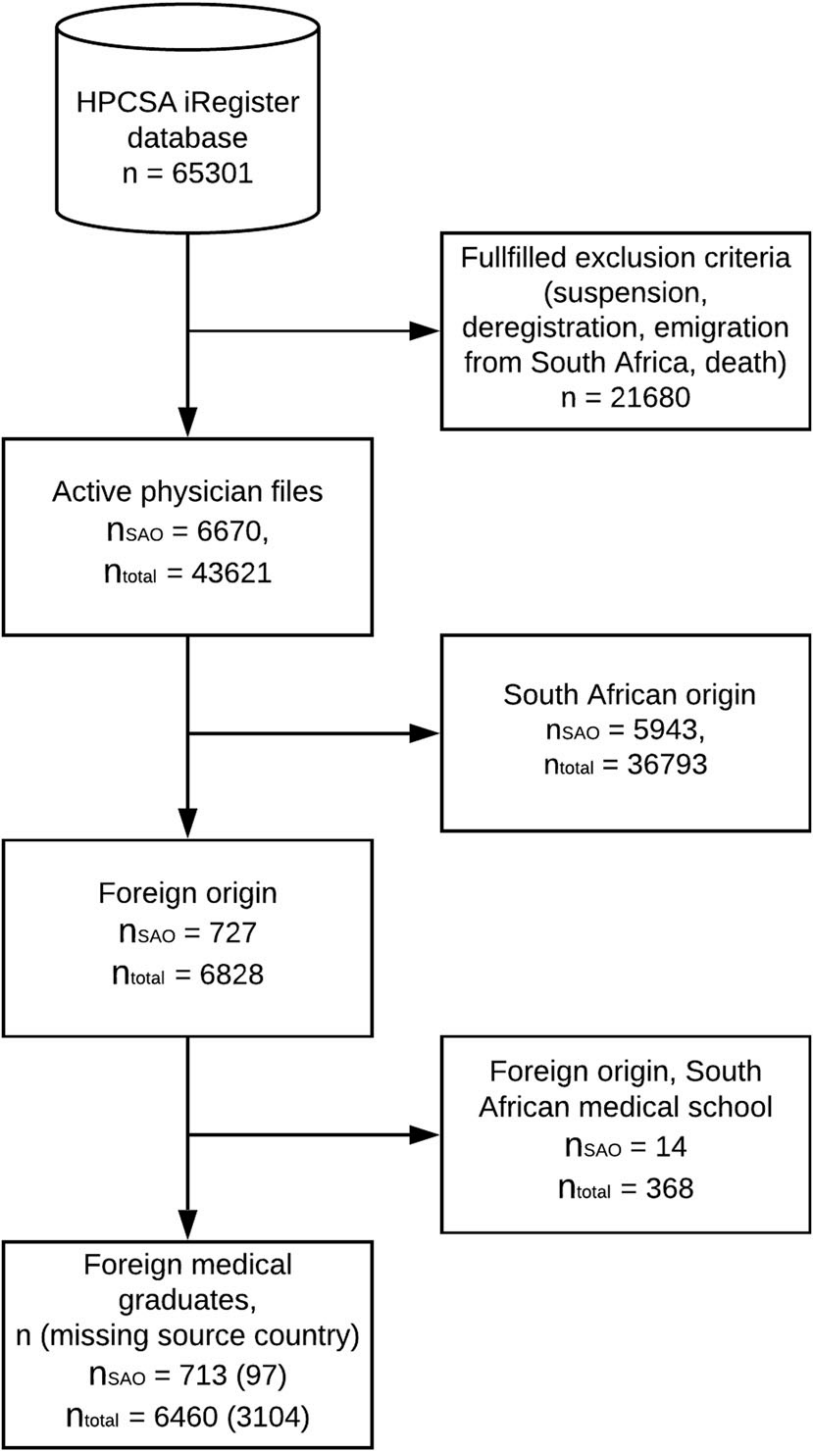
Inclusion of South African foreign medical graduates and the subgroup of surgeons, anesthesiologists, and obstetricians. The HPCSA iRegister database includes South African physicians from the Health Professions Council of South Africa [21]. *n*_total_ = number of physicians, *n*_SAO_ = number of surgeons, anesthesiologists, and obstetricians

We included all physicians registered to practice in South Africa (Fig. 1). Data on physicians in South Africa were obtained from the public iRegister database provided by the Health Professions Council of South Africa (HPCSA) [21]. We considered SAO specialists as any physician licensed within surgery, pediatric surgery, ophthalmology, plastic and reconstructive surgery, orthopedics, otorhinolaryngology, urology, neurosurgery, cardiothoracic surgery, anesthesiology, or obstetrics and gynecology. We excluded all non-active physicians. Reasons for exclusion from the calculations on SAO specialists in South Africa included suspension, deregistration, emigration from South Africa, and death. Data were for 2016, corresponding to the 2016 update of the WHO surgical workforce database, the last year for which complete worldwide data are available [22, 23].

### Foreign SAO specialists in South Africa

The HPCSA database contains information on the specialty of health practitioners, medical school, foreign origin, and postgraduate credentials. We defined the source country as the country of earliest available medical qualification. A foreign medical graduate was defined as a non-South African, with a medical degree from outside South Africa.

### South African SAO specialists working abroad

Data on SAO specialists from South Africa working abroad were retrieved from the national databases of 14 HICs [3], and the included countries were Australia, Austria, Canada, Estonia, Finland, Ireland, Israel, New Zealand, Norway, Slovakia, Slovenia, Sweden, The United Kingdom, and the United States. These were the countries for which data were available, though we note they also capture the vast majority of migration from South Africa [19]. The specialist designation was defined according to the regulations of the destination country, and South African physicians were identified by their medical school graduation.

### Outcomes and statistical analysis

The primary outcome was the number of foreign SAO specialists in South Africa and the number of South African SAO specialists working abroad. Two proportions were calculated to contextualize these numbers. (1) From the perspective of receiving country, we calculated the South African dependency on SAO specialists from low- and middle-income countries (LMICs) by dividing the number of foreign medical graduates from LMICs by the total number of South African SAO specialists. (2) From the perspective of source country, we calculated the emigrated proportion of SAO specialists from each source country, defined as $A/\left( {A + B} \right)$, where A is the number of SAO specialists from a source country who have emigrated, and B is the number of SAO specialists remaining in the source country. Finally, we calculated the correlation between the proportion of emigrated SAO specialists in a source country with gross national income per capita and the density of SAO specialists, using linear regression.

Information on source country was missing for a subset of foreign medical graduates in South Africa. These were assigned an origin country based on the proportion in the observed data, i.e., a missing completely at random assumption was made. Calculations were performed in R (version 4.1.0, R core team, Vienna, Austria, 2021).

### Auxiliary data sources

Data on the total number of SAO specialists in the source countries were obtained from the WHO surgical workforce database [20, 24], which in turn sources data (in order of preference) from governmental sources, professional bodies, WHO/OECD/Eurostat databases, scientific publications, and other reports. Where data were not available, estimations based on a multiple imputation prediction model were used [22]; estimated number of SAO specialists from the Democratic People’s Republic of Korea were excluded due to data quality concerns. Data on gross national income per capita in purchasing power parity and population were obtained from the World Bank World Development Indicators database [24]. Countries were categorized by World Bank Group income classification and regions were categorized by WHO classification [17, 24].

### Ethical considerations

The project was exempt from full ethical review by the institutional review board at Boston Children’s Hospital (IRB-P00024135).

## Results

**Fig. 2:**
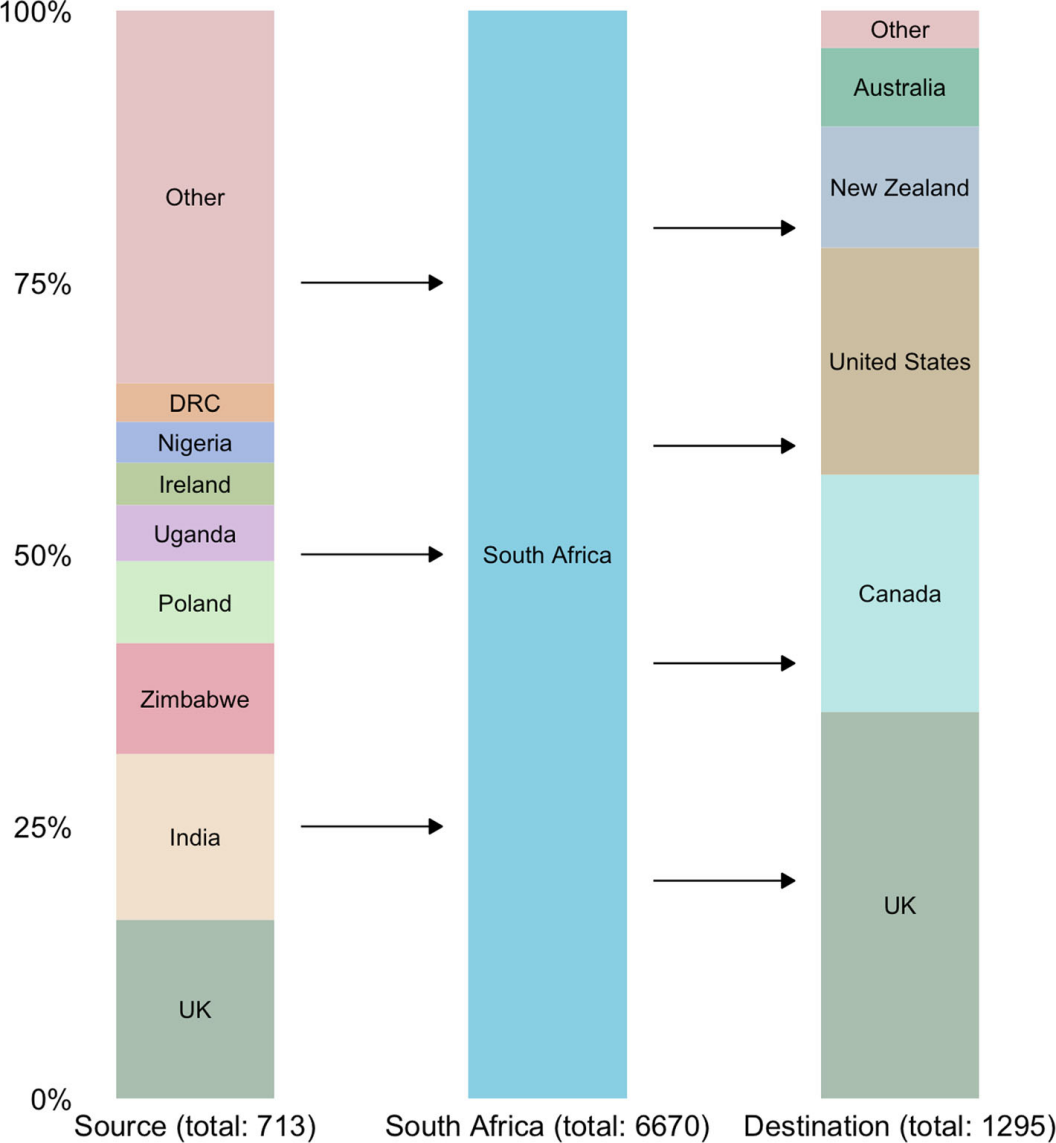
The leading source and destination countries for surgery, anesthesia, and obstetric specialists, who have migrated in and out of South Africa. In addition to the 8 origin countries displayed here, an additional 49 countries sent at least 1 SAO specialist to South Africa, although no country in the “other” category sent more than 25 SAO specialists

We identified 43,621 physicians in South Africa, of whom 3685 were surgeons, 1749 were anesthesiologists, and 1236 were obstetricians, corresponding to a total of 6670 (15%) SAO specialists, or 12.1 per 100,000 population (Fig. 1). In addition, 1295 SAO specialists (16%) in the 14 assessed high-income countries originated from South Africa (Fig. 2).

**Fig. 3:**
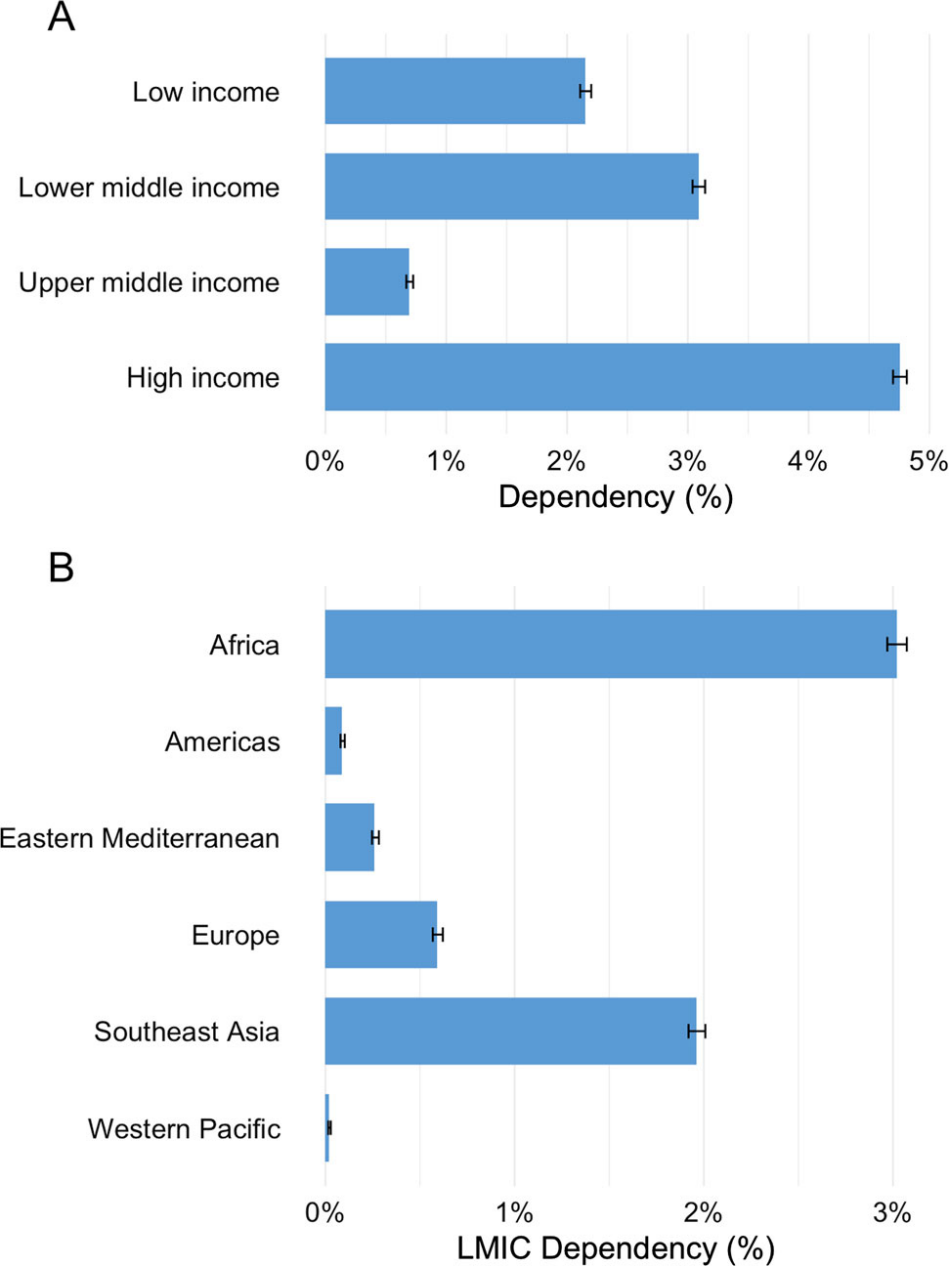
**a** and **b**. The South African dependency on foreign medical graduates in surgery, anesthesia, and obstetrics. **a** by World Bank income group, **b** by WHO region, including only low- and middle-income source countries. Dependency is the number of foreign medical graduates divided by the total number of physicians

**Fig. 4:**
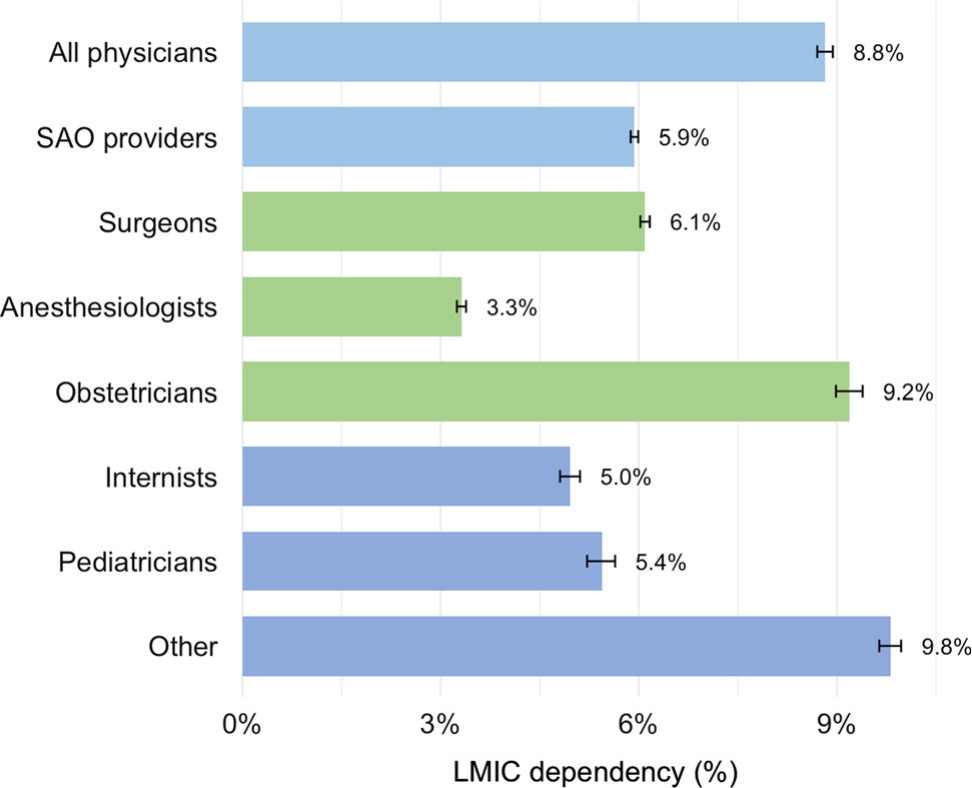
The South African dependency on foreign medical graduates from low- and middle-income countries, by specialty. Dependency is the number of foreign medical graduates divided by the total number of physicians in each specialty

Of the 6670 SAO specialists in South Africa, a total of 713 (11%) were foreign medical graduates and 396 (5.9%) were from an LMIC, mostly from Africa (201, 3.0%) and Southeast Asia (131, 2.0%) (Fig. 3). The South African dependency on foreign medical graduates from LMICs was lower for SAO specialists compared to physicians in general (5.9% and 8.8%, respectively) and, among SAO specialists, the dependency was highest for obstetricians (9.2%) and lowest for anesthesiologists (3.3%) (Fig. 4).

**Fig. 5:**
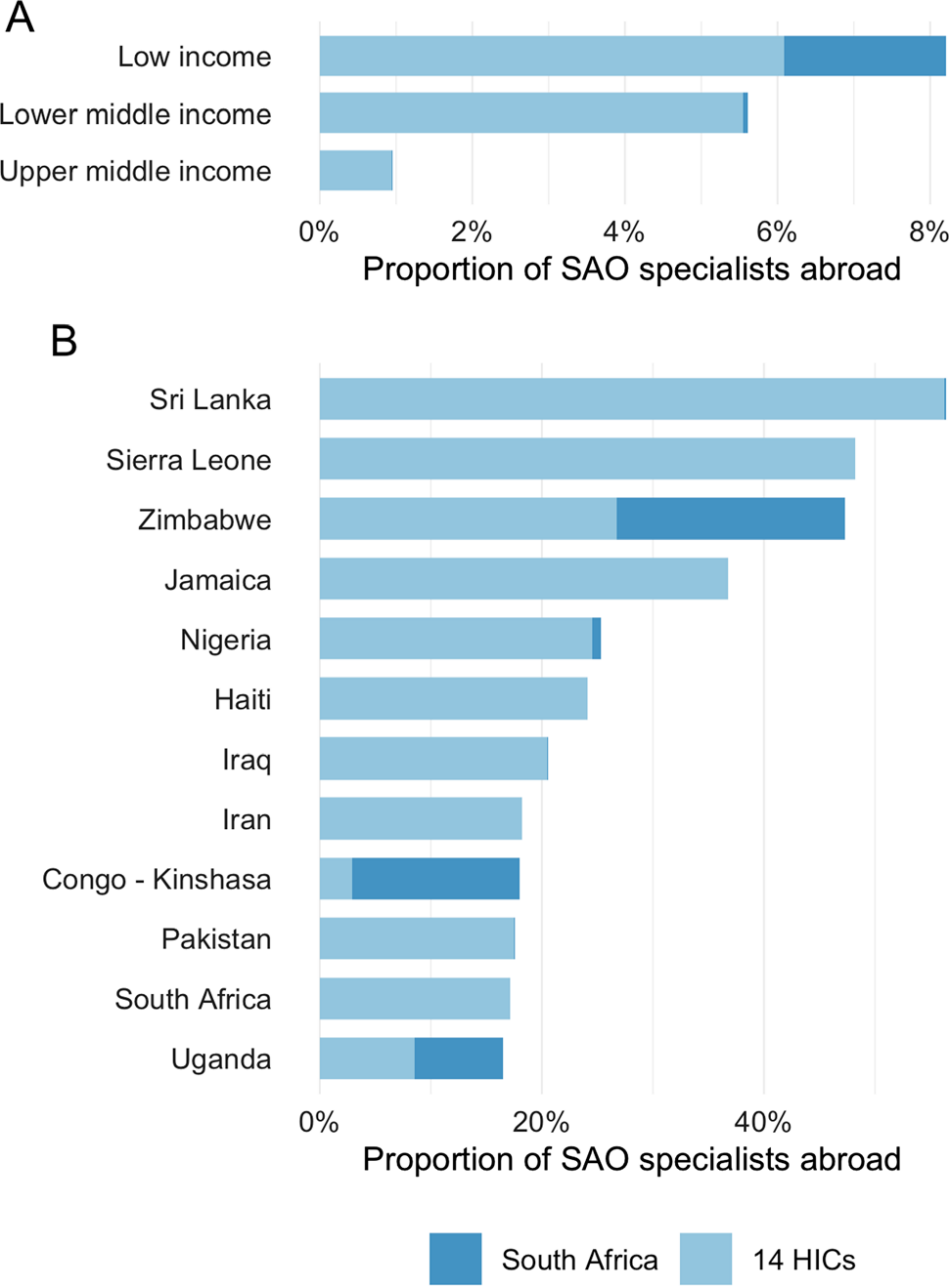
**a** and **b** The proportion of SAO specialists abroad in South Africa and 14 high-income countries (HICs), **a** by World Bank income group, and **b** by country, displaying the top countries. The proportion was defined as *A/(A* + *B)*, where A is the number of SAO specialists who have emigrated and B is the number of SAO specialists remaining in the source country or region

**Fig. 6:**
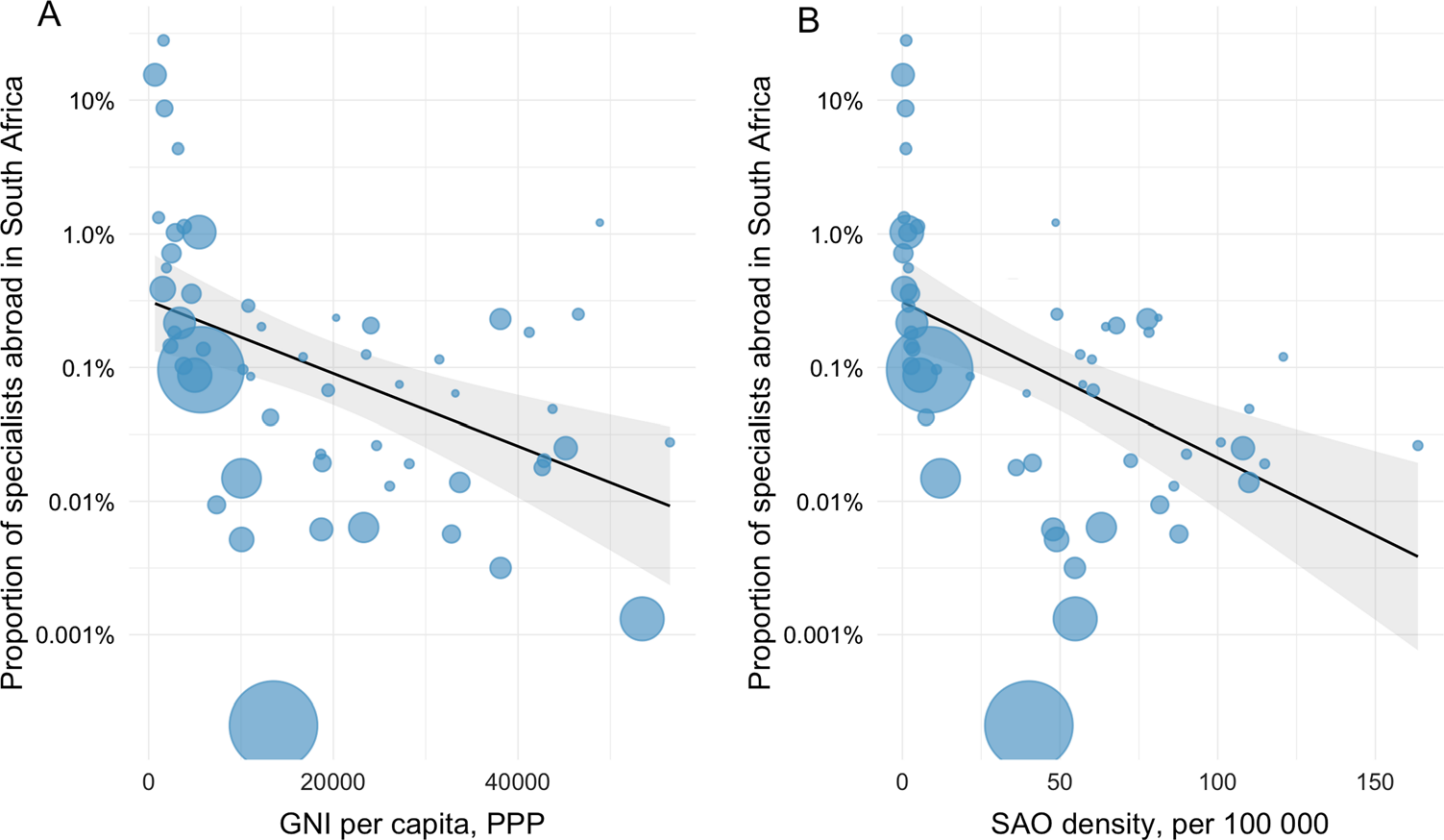
**a** and **b**. The correlation between the proportion of surgery, anesthesia, and obstetric specialists abroad in South Africa and **a** gross national income per capita, **b** and the remaining density of SAO specialists per 100,000 population. The proportion was defined as *A*/(*A* + *B*), where A is the number of SAO specialists from a source country who have emigrated to South Africa, and B is the number of SAO specialists remaining in the source country. Bubble sizes represent source country population size

Figure 5 displays the proportion of SAO specialists from individual countries and by income class which have migrated to HICs or to South Africa. Of all SAO specialists originating from low-income countries, a total of 8.2% worked in any of the included destination countries, 6.1% in one of the 14 assessed high-income countries, and 2.1% in South Africa. For SAO specialists originating in lower-middle income countries 5.5% worked in an HIC and 0.06% in South Africa, and for upper-middle income countries 0.94% worked in an HIC and 0.005% in South Africa (Fig. 5). For every 1000$ increase in the gross national income, there was a 6.3% (95% CI 2.9–9.6%, *p* < 0.001) decrease in the proportion of specialists abroad in South Africa, and for every increase in surgeon, anesthesiologist, and obstetrician density of 1/100 000, there was a 2.8% (95% CI 1.5–4.1%, *p* < 0.001) decrease in the proportion of specialists abroad in South Africa (Fig. 6).

## Discussion

In this study, we provide a mobility analysis of the SAO specialist workforce in South Africa. Sixteen percent of South African SAO specialists currently work in a high-income country, and 6% of the SAO specialists currently in South Africa originate from another LMIC. Given the severe shortage of SAO providers in sub-Saharan Africa, many neighboring countries have a large diaspora of SAO specialists currently working in South Africa.

The maldistribution of SAO specialists in the world is exacerbated by specialist migration to more affluent regions. Our study shows that South Africa is an important hub for physician migration into and out of sub-Saharan Africa. It is possible that the large emigration from South Africa has created a physician shortage that in turn is partly replaced with migrants from other LMICs and that this in turn impacts source countries with an already insufficient supply of SAO providers. For example, Zimbabwe, the Democratic Republic of the Congo, and Uganda have a particularly high proportion of SAO specialists currently working in South Africa, especially when considering that these countries also lose large numbers of medical graduates to high-income countries. The South African dependency on SAO specialists originating from LMICs was 5.9%, as compared to a previous study which reported 9.7% for high-income countries, with a range of 0.3% to 15.6% [3].

The effects of physician migration are certainly not all negative, and it is reasonable to also underline the potential for “brain gain.” Emigration can bring benefits to the source country in several ways. First, the temporary or permanent return of gifted individuals having spent time abroad can bring valuable knowledge and skill sharing [2, 25, 26], and considerable interaction between the “surgical diaspora” and the source countries has been described [27]. SAO trainees or fellows temporarily attending training programs abroad can in many circumstances be an appropriate strategy to mitigate national shortages of SAO specialists, with South Africa in particular suppling training opportunities for sub-Saharan African SAO trainees [28] Second, many emigrated physicians send home remittances, which in some cases are quite considerable [6, 25, 26]. Third, the opportunity for migration to more affluent circumstances can potentially serve as an incentive for education [25]. Emigration also poses a chance for the individual to gain better prospects for career opportunities, postgraduate training opportunities that may not exist in the home country, better working conditions, and higher salary [6]. Indeed, a report by the South African Migration Programme found that a significant number of South African physicians had worked temporarily in other countries, primarily in Western countries, often to pay off student loans or gain valuable skills [29]. Finally, the considerable size of this “surgical diaspora” in relation to the remaining workforce in some source countries could potentially serve as a valuable pool of temporarily or permanently returning highly trained specialists, either providing clinical services directly or teaching younger colleagues. Return migration from HIC to South Africa has led to a decreased net emigration especially in the last two decades [11], with time-limited employment in HICs and social and environmental factors in South Africa being the main driving forces for return migration [30]. It should also be noted that drivers of migration are complex and multifaceted, often divided into push and pull factors. Push factors are motivations for migration in the source country, such as poor working conditions and remuneration. In particular, a lack of stable employment opportunities in the home country represents a major push factor for primary migration and hinders return migration, both for South Africa [30] and for other sub-Saharan African countries [31, 32]. Pull factors are motivations for migration in the receiving country, such as better opportunities for career development and training [15, 26, 33]. Some qualitative studies have suggested push factors to be more important than pull factors.[13, 33–35].

The large-scale migration of skilled health workers has stirred a vehement ethical debate in the international community [6, 13, 36, 37], bringing into opposition the right of the health worker to free mobility with the right to health for the population of the source country. Adding to the controversy, the countries with the most severe shortage of skilled health-care workers are often the most affected, as evident by the negative correlation between SAO specialist density and emigrated proportion in our data. Since medical education is often subsidized by the state, the migration of physicians also constitutes a financial loss for sending countries, on the order of several billion USD [38, 39]. Several important steps need to be taken to minimize any compromise of either principle. Some authors make a distinction between active and passive recruitment, where the former can include recruiting elements such as advertisements and information sessions encouraging and promoting migration [10, 36]. Such active practices are often discouraged [36, 40, 41]. Policies that increase worker retention by decreasing the effects of push factors can circumvent the ethical dilemma by instead working to increase job satisfaction in the source countries [26, 36, 42]. Other important measures include careful risk–benefit analysis, transparency in hiring and remuneration practices, and supporting the health-care capacity building of developing countries [40]. In 2010, after determined advocacy from some African countries [6], all member states of the United Nations adopted the non-binding “WHO Code of Practice on the International Recruitment of Health Workers,” which lays a framework for the ethical recruitment practices of health workers [9, 40]. Per the Global Strategy on Human Resources for Health [43], adopted by the 69th World Health Assembly through resolution WHA69.19, member states should strive to halve their dependency on foreign medical graduates by 2030, and to gather and track relevant data to enable policy to be driven by evidence [43, 44].

It should be noted that South Africa in particular has attempted to reduce its recruitment of physicians from other LMICs, especially from the Southern African Development Community area [12, 13]. They have also signed memoranda of agreement with some African countries not to employ their citizens after graduation from medical training in South Africa [13, 45]. The efficiency of these policies has been questioned; some report ready movement into South Africa despite these policies [13, 19], while some argue the policies have merely redirected the flow of migrants to Northern countries [15]. South Africa has also instituted training programs in SAO specialties for foreign medical graduates, known as supernumerary registrars [46]. These supernumerary registrars are expected to return after training to their country of origin to provide specialized care in underserved regions, and indeed are often also financed by their country of origin. A change in regulations regarding the expectation for supernumerary registrars to return to their country of origin upon training completion would substantially increase migration to South Africa from other African countries.

Since longitudinal data were not available, we were not able to draw any conclusions on the yearly flow of SAO specialists which would enable us to evaluate immigration policy changes, both to and from South Africa. In particular, it is possible that policies enacted by South Africa as outlined above have lowered the influx of physicians from other African countries, and that many of the current SAO specialists migrated before current policies were put in place. A longitudinal analysis of South African physician immigration between 2000 and 2014 found an increase in immigration during that period [19], while emigration from South Africa to high-income countries has decreased over the past decades, with the proportion of physicians registered abroad decreasing from a peak of 34% in 2005 to 22% in 2017 [11]. However, data are not available broken down by specialty. Future studies may also elucidate whether the COVID-19 pandemic affected physician migration. There is a paucity of data and it would be valuable if future studies focus on the temporal patterns of SAO specialist migration. One potential avenue forward would be to include not only the SAO density, but also key metrics affecting SAO density such as graduation, retirement, and migration, in the measurement and evaluation strategies of National Surgical, Obstetric and Anesthesia plans [47].

It is important to consider some limitations of our study. Similar to previous studies, it was not possible to distinguish at which point in the training the SAO specialists migrated, i.e., before or after postgraduate training. National databases may contain SAO specialists who are no longer actively practicing in the said country but have not yet deregistered, with practices differing between countries, although deregistration upon return migration to South Africa is commonplace [11]. Some physicians migrating to a new country may fail to obtain a license and go into some other occupation, causing an underestimation [12, 14]. For a proportion of physicians, data were not available on source country, and we adjusted the estimates proportionally to account for the missing data. If, in fact, there is a systematic bias to the way the data are missing, our results could under- or over-estimate the number of physicians originating in individual countries. Due to data limitations, our study did not include non-physician SAO providers, who play an important role in the provision of surgical care in some countries [48]. The study also does not capture physicians currently in surgical training, who may be especially mobile.

## Conclusion

South Africa is a major hub for physician migration, and 6% of SAO specialists in South Africa were medical graduates from other low- or middle-income countries, whereas 16% of SAO specialists from South Africa have migrated to high-income countries. Eight percent of SAO specialists originating in low-income countries have emigrated abroad, a fourth of which have migrated to South Africa.
